# A new natural hybrid of *Iris* (Iridaceae) from Chongqing, China

**DOI:** 10.3897/phytokeys.174.62306

**Published:** 2021-03-05

**Authors:** Yue-e Xiao, Feng-yang Yu, Xiao-feng Zhou

**Affiliations:** 1 Research Center, Shanghai Botanical Garden/Shanghai Engineering Research Center of Sustainable Plant Innovation, Shanghai 200231, China Shanghai Botanical Garden/Shanghai Engineering Research Center of Sustainable Plant Innovation Shanghai China; 2 Chongqing City Flower Fragrance Horticulture Limited Company, Chongqing 402764, China Chongqing City Flower Fragrance Horticulture Limited Company Chongqing China

**Keywords:** chloroplast DNA, Chongqing, *Iris* × *ampliflora*, Section *Lophiris*

## Abstract

A newly discovered natural hybrid, Iris×ampliflora Y.E. Xiao, F.Y. Yu & X.F. Chen (Iridaceae: subgenus LimnirissectionLophiris) from Chongqing, China, is described and illustrated. This hybrid is morphologically similar to *I.japonica* Thunb. and *I.wattii* Baker, but can be distinguished by its giant leaves and large purple flowers. Phylogenetic trees based on cpDNA data support the separation of I.×ampliflora from other closely related species in the section Lophiris. According to its morphological features, molecular systematic evidence and chromosome data, we speculate that I.×ampliflora [31 chromosomes] likely is a new hybrid between *I.japonica* [2*n* = 32] and *I.wattii* [2*n* = 30].

## Introduction

*Iris* L. is the largest genus in family Iridaceae with up to 280 species that are mainly distributed in temperate regions of the Northern Hemisphere (Goldblatt and Manning 2008). The characteristics of iris flowers are petaloid-style branches, two obvious perianth whorls, and floral tubes with nectaries (Goldblatt and Manning 2008). Irises produce showy flowers and are well-known and popular ornamental plants worldwide. To gain more cultivars for gardens, many crosses between *Iris* species have been attempted and achieved success by artificial hybridization (Hu and Xiao 2012; Yu et al. 2017). Natural hybrids between sibling species do occur in the genus *Iris*, for example in some species within subgen. Limniris in North America (Burke et al. 1998). However, there is no natural hybrid of *Iris* reported in East Asia.

The outer sepals of irises are equipped with raised beards, crests, signal patches, and midveins, which probably have significance for pollination (Sapir et al. 2005; Guan et al. 2009; Xiao et al. 2019). The sepal crest is an important character in the taxonomic delineation of higher ranks of *Iris*. Dykes (1913) first placed six rhizomatous species in sect. Evansia. Lawrence (1953) changed Evansia to the subsection level. Rodionenko (1987) elevated subsect. Evansia to subgen. Crossiris. Mathew (1981) elevated subsect. Evansia of Lawrence to section level, namely sect. Lophiris, placing the section within subgen. Limniris. sect. Lophiris contains 11 species, most of which are distributed in Eastern Asia, apart from three species in North America (*I.cristata* Solander, *I.lacustris* Nuttall, and *I.tenuis* S. Watson) (British Iris Society Species Group 1997; Zhao et al. 2000). Mathew’s classification was used in this study.

During field work in Chongqing, we found an interesting specimen originally from the Qingyang Town in Fuling District, Chongqing City, China. Our observations show that it is morphologically similar to *I.japonica* Thunb. and *I.wattii* Baker with a yellow and irregularly toothed crest on the outer segments. However, its morphological features differ markedly from those of all known species in sect. Lophiris described by Mathew (1981). We have observed the situation concerning seed set since 2014 when the species was initially introduced to the conservation nursery of Shanghai Botanical Garden. It is sterile with no seed production, which indicates its hybrid origin.

This study was undertaken to assess the status and parentage of the new hybrid by morphological surveys, phylogenetic and chromosome data. The Qingyang collection is a large evergreen plant with large attractive flowers, and it can adapt to warm, wet, and full-sun or partly shady environmental conditions. It has potential uses in breeding and landscaping. We formally publish its description here with the aim of better understanding and utilization of the remarkable morphological divergence of Iris species in subgenus Limnirissect.Lophiris.

## Materials and methods

### Morphological surveys

Living specimens and vouchers of the new hybrid were examined and compared with four species of sect. Lophiris (*I.confusa* Sealy, *I.japonica* Thunb., *I.tectorum* Maxim. and *I.wattii* Baker) in Southern China using measurements and descriptions of the main characteristics. Species from the conservation nursery of Shanghai Botanical Garden from mid-March and late-April in 2019 were compared. Eight or ten randomly chosen individuals of each taxon were used for the morphometric surveys (Table 1). Meanwhile, herbarium sheets (CDBI, CSH, IMC, IBSC, LBG, KUN, PE) and type descriptions of *I.confusa*, *I.japonica*, *I.milesii* Baker ex Foster, *I.tectorum* and *I.wattii* were compared with the new hybrid.

**Table 1.** Differences between Iris×ampliflora, *I.confusa*, *I.tectorum*, *I.wattii* and *I.japonica* in the conservation nursery of Shanghai Botanical Garden.

Species		*I.tectorum*	*I.confusa*	*I.wattii*	I.×ampliflora	*I.japonica*
		(n = 10)	(n = 10)	(n = 8)	(n = 10)	(n = 10)
Aerial stem Length		No	67.2 ± 19.9a	30.3 ± 9.3b	27.0 ± 2.7b	12.3 ± 2.7c
Leaf	Waxy	No	Yes	No	No	Yes
	Longitudinal veins	Clearly	Clearly	Clearly	Clearly	Clearly
	Texture	Wrinkled	Smooth	Wrinkled	Wrinkled	Smooth
	Length	49.5 ± 3.5c	54.8 ± 3.8b	70.1 ± 9.2a	74.2 ± 6.4a	48.2 ± 2.9c
	Width	2.9 ± 0.2d	5.3 ± 0.6b	4.3 ± 1.0c	6.6 ± 0.8a	3.8 ± 0.7c
Flower	Flowering-stem	1–2 branches	2–4 branches	5–7 branches	7–10 branches	5–12 branches
	Color	Bluish violet	Pale reddish purple	Bluish violet	Violet	Violet / Bluish violet
	Size (in diam.)	9.2 ± 1.1b	5.1 ± 0.4d	7.3 ± 0.4 c	12.5 ± 0.5a	4.8 ± 0.4d
	Crest	White	Yellow	Yellow	Yellow	Yellow
	Anthers	White	White	Yellow	White	White
Chromosome number		28	30	30	31	28, 30, 31, 32, 33, 34, 35, 54 and 55
Distribution		Subtropical and temperate zone of China	Chongqing, Sichuan, Xizang, Yunnan [NW India]	Chongqing, Sichuan, Xizang, Yunnan [NE India, Myanmar].	Chongqing	Subtropical and temperature zone of China [Japan]

Note: n, sample number.

### Phylogenetic analyses

We collected samples of five species / hybrid (*I.confusa*, *I.japonica*, *I.tectorum*, *I.wattii*, and Qingyang collection) of sect. Lophiris, and three species (*I.anguifuga* Y.T. Zhao, *I.henryi* Baker and *I.proantha* Diels) of section Limniris and *Irisdomestica* (L.) Goldblatt & Mabb. to construct phylogenetic trees based on cpDNA data. The samples of the new hybrid were collected from the type locality. Other iris samples were collected from the conservation nursery of the Shanghai Botanical Garden.

Total genomic DNA was extracted from each sample (30 mg dried leaves) using a DNA Plant Kit (Tiangen, Shanghai, China) according to manufacturer’s instructions. The extracted DNAs were dissolved in 100 μl TE buffer for storage. We amplified and sequenced part of the *mat*K gene (Wilson 2004) and *ndh*F gene (Shaw et al. 2007) (Table 2). Each PCR mixture (50 μl) contained ddH_2_O, 1 × buffer (Mg^2+^ free), 2.5 mM MgCl_2_, 2.5 mM each dNTP, 0.5 μM each primer, 2 U Taq polymerase (Sangon, Shanghai, China), and 20 ng DNA. The PCR reactions were conducted on a Mastercycler pro Thermal Cycler (Eppendorf, Hamburg, Germany). The procedure was performed with initial denaturing for 5 min at 94 °C followed by 35 cycles of 30 sec at 94 °C (denaturing), 30 sec at 50 °C (annealing), and 45 sec at 72 °C (elongation), and final extension for 10 min at 72 °C. After checking products by electrophoresis on a 1.2% agarose gel, the purified products were bi-directionally sequenced by standard methods on the ABI 3731 automated sequencer (Applied Biosystems, Foster City, CA, USA). The sequences of these irises have been deposited in GenBank (see accession numbers in Appendix 1: Table A1. For the cpDNA datasets, the CLUSTALW program combined with manual adjustment was used for multiple alignments of all sequences (Thompson et al. 1994). We used three phylogenetic methods (i.e., Bayesian inference, maximum parsimony, and maximum likelihood) to analyze the alignments. We conducted the Bayesian inference analysis with MrBayes v.3.2.6 (Ronquist and Huelsenbeck 2003), and the maximum parsimony and maximum likelihood analyses using PAUP v.4.0b10 (Swofford 2002).

**Table 2.** Primers used in this study.

Gene name	Primer	Sequence (5′ to 3′)
*mat*K	*mat*K-3914F	ATCTGGGTTGCTAACTCAATGG
(Wilson 2004)	*mat*K-1235R	GGAGTGGGGTATTAGTATA
	*mat*K-1176F	CTATTCATTCCATTTTTCCT
	*mat*K-*trnK*2R	AACTAGTCGGATGGAGTAG
*ndh*F	*ndh*F-*pair*1	ATGGAACA(GT)ACATAT(CG)AATATGC
(Shaw et al. 2007)	*ndh*F-1201ir	GGAATACCACAAAGAGAAAGTGTACCT
	*ndh*F-972i	GTCTCAATTGGGTTATATTATG
	*ndh*F-2210R	CCCCCTA(CT)ATATTTGATACCTTCTCC

### Chromosome number analyses

To determine the chromosome number of the new hybrid from somatic cells. Root tips (1–1.5 cm in length) were collected and washed with distilled water and immersed in 0.002 mol/L 8-hydroxyquinoline with dark pretreatment for 2–2.5 h. These roots then were fixed in Carnoy solution (volume ratio: 95% ethanol: acetic acid = 3:1) at 4 °C for 2–3 h. The fixed roots were dissociated in 5 mol/L hydrochloric acid for 8–10 min and washed with distilled water, then stained with carbol fuchsin and squashed on glass slides. Finally, the samples were observed and photographed using a Motic BA400 optical microscope. More than 20 cells were observed to determine the number of chromosomes for each specimen examined.

## Results

### Morphological comparisons

**Figure 1.**
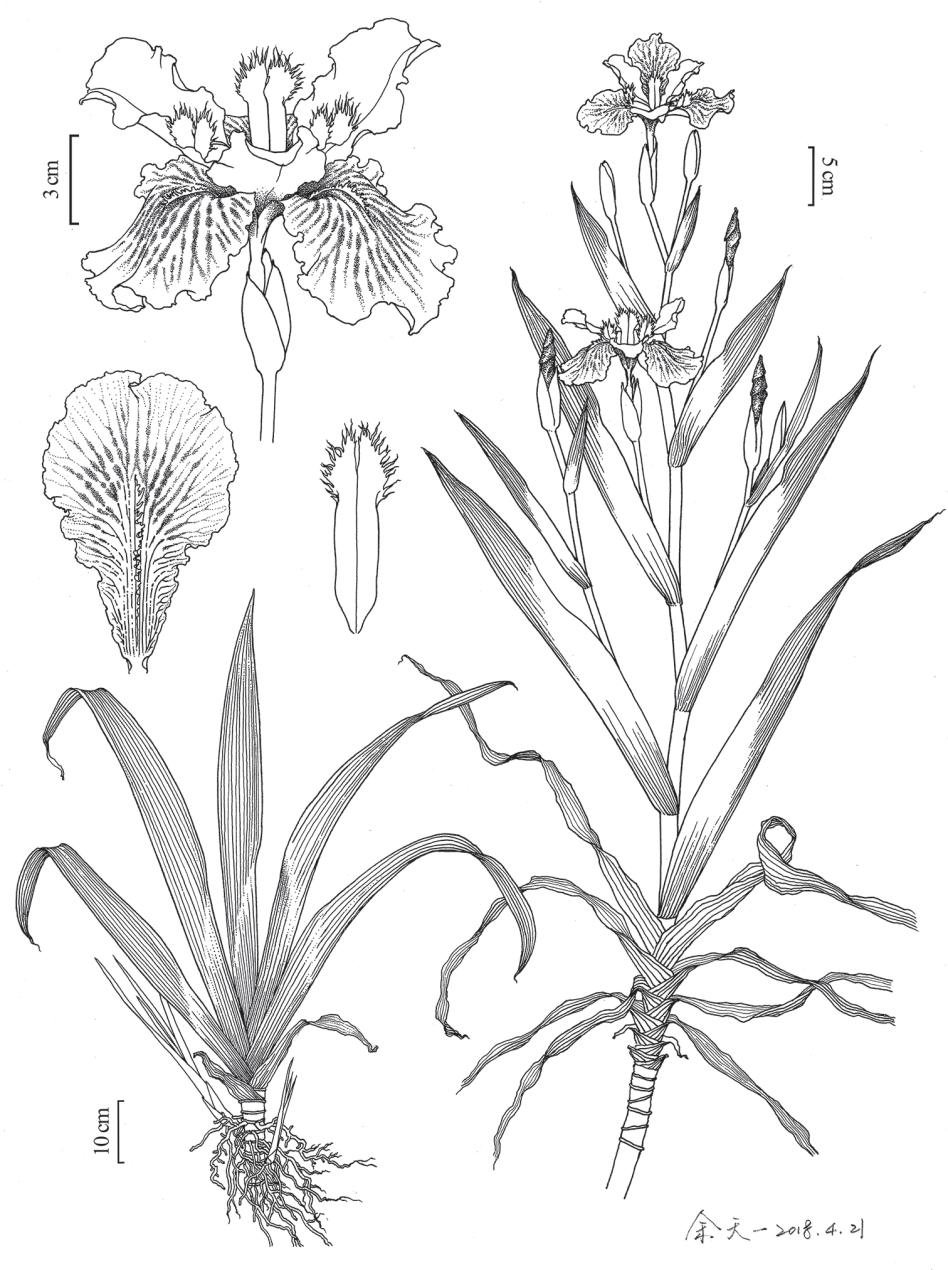
Line drawings of Iris×ampliflora based on photos and the type specimens (Drawn by Tian-Yi Yu).

**Figure 2.**
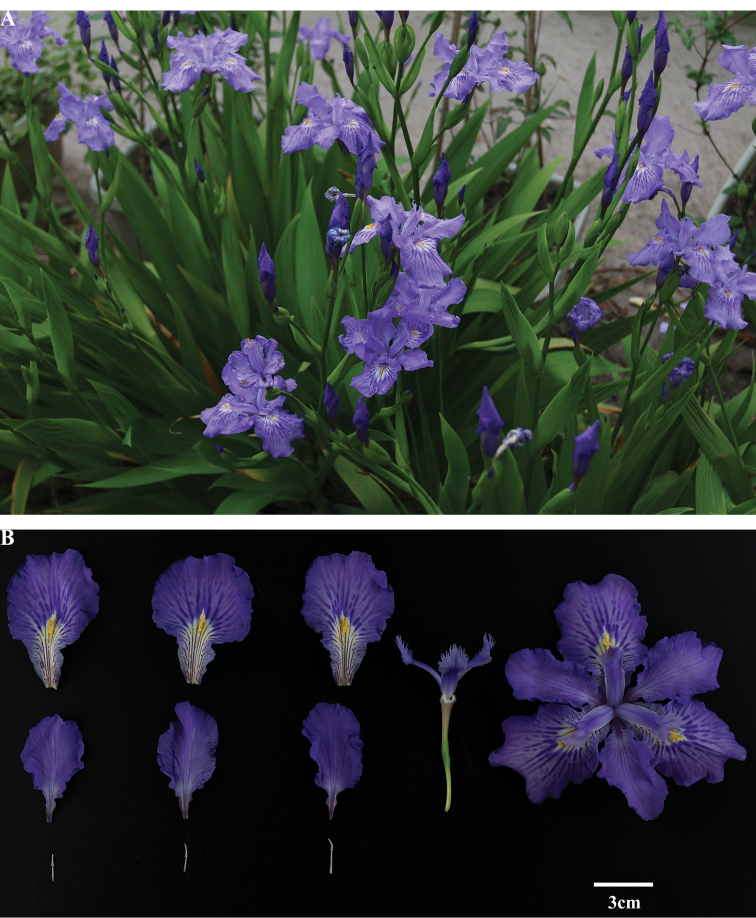
**A**Iris×ampliflora in flower **B** flower anatomy of Iris×ampliflora.

The Qingyang collection is morphologically similar to the species of section Lophiris, *I.japonica* which has 5–10 branches of the flowering stem, a yellow crest on the outer sepals, and is the most common species of *Iris* in Southern China (Zhao et al. 2000). However, the Qingyang collection has several characteristics that distinguish it from *I.japonica*, *I.confusa*, *I.milesii*, *I.wattii* and *I.tectorum*, including plant larger flowers, larger leaves, and other features as described in Table 1. The flowering stem of this specimen has 7–10 branches and an aerial stem (mean length = 27.0 ± 2.7 cm, n = 10) (Fig. 1). Compared with other species in sect. Lophiris, the Qingyang collection has larger leaves (mean length = 74.2 ± 6.4 cm, n = 10; mean width = 6.6 ± 0.8 cm, n = 10) and larger purple flowers (mean diameter = 12.5 ± 0.5 cm, n = 10) (Fig. 2).

### Phylogenetics

Among eight individuals of different species / hybrid, there were 318 variable sites, 187 singleton variable sites, and 131 parsimony informative sites across 4779 bp aligned positions of two cpDNA fragments. There were 22 and 39 mutations between the sequences of *I.japonica* / *I.wattii* and the new hybrid, respectively.

**Figure 3.**
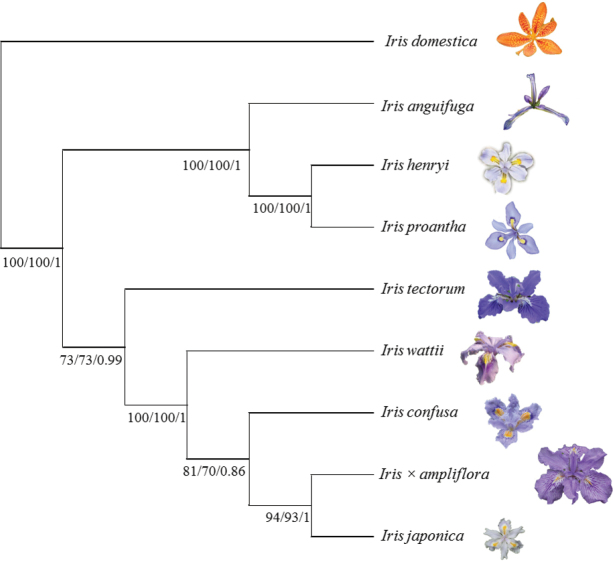
Phylogenetic relationships of Iris×ampliflora based on two plastid DNA fragments. Numbers around nodes are Bayesian posterior probabilities and bootstrap percentages (PP /BS_MP_/BS_ML_). (Pictures of flowers are relatively proportional sizes among different *Iris* species).

In the molecular tree based on cpDNA data, the sampled new hybrid was resolved as sister to the sample of *I.japonica* (Fig. 3). In Bayesian, maximum parsimony, and maximum likelihood trees based on cpDNA data, this specimen clustered into a clade with *I.japonica* (Fig. 3). The inter-clade sequence difference (*I.japonica* vs. *I.wattii*, 0.11) was 22 times greater than the intra-clade sequence differences (the new hybrid vs. *I.japonica*, 0.05).

### Chromosome number

**Figure 4.**
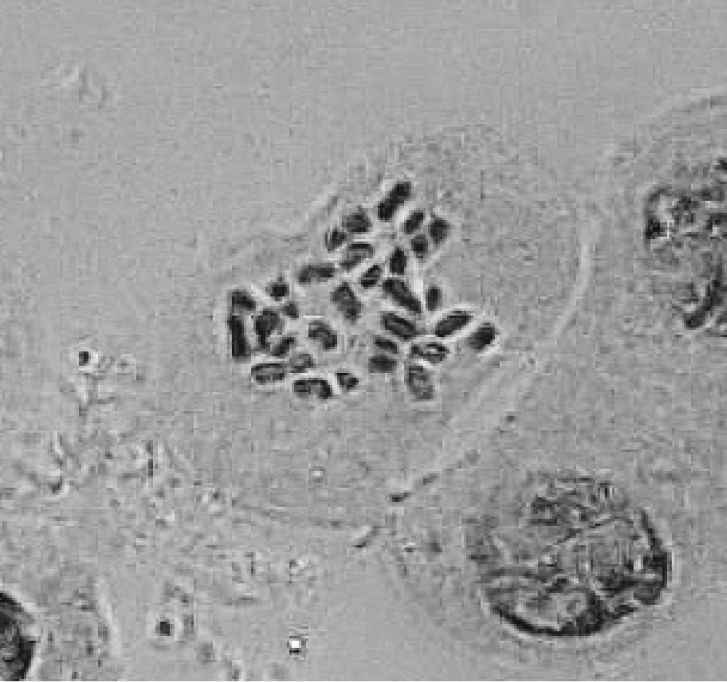
Mitotic metaphase chromosomes of Iris×ampliflora [number of chromosomes, 31].

Cytological study indicated that the Qingyang collection has 31 chromosomes (Fig. 4). No evidence of chromosome pairing could be examined and it is unknown if it functions as a diploid.

### Taxonomy

#### Iris × ampliflora

Taxon classificationPlantaeMantodeaTarachodidae

Y.E. Xiao, F.Y. Yu & X.F. Chen nothosp. nov.

B2DF36F7-DC0C-5D58-B02A-5ACCE9B11ECB

**Figure A1.**
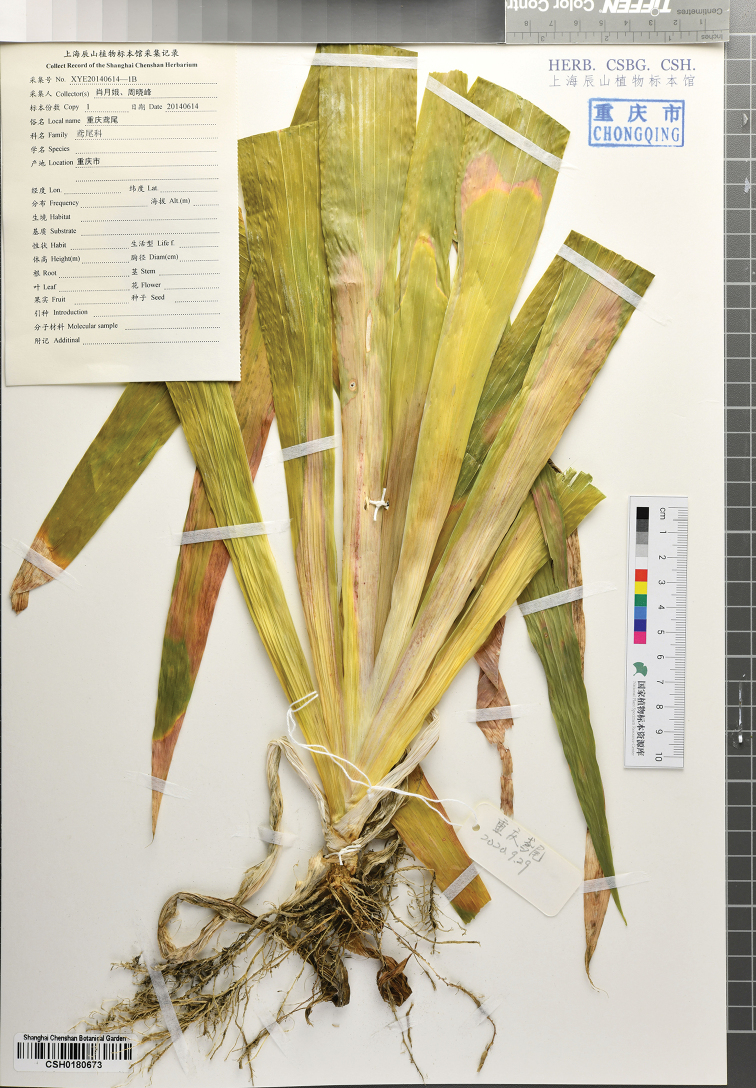
The photo of holotype of Iris×ampliflora.

Figures 1 , 2 , Appendix 2 : Figure A1

##### Diagnosis.

Morphologically similar to *I.japonica*, the new hybrid differs in having 7–10 branches and an aerial stem (25–29 cm compared to 10–15 cm), larger leaves (length and width, 68–80 cm and 6–7 cm compared to 45–51 cm and 3–4.5 cm) and larger purple flowers (diam., 11–13 cm compared to 4–5 cm).

##### Type.

Shanghai Botanical Garden, grown from collection from the Qingyang Town of Fuling District, Chongqing, 14 June 2014, *Y.E. Xiao XYE20140614* (holotype: CSH-0180673!; isotypes: CSH!). A photo of the holotype is shown in Appendix 2: Figure A1.

##### Description.

*Rhizomes* creeping, thick, ca. 1.5–2 cm in diam. *Overall plant* up to 85.4–125.5 cm. *Stem* ascending upright, 24–31 cm. *Leaves* mainly in basal fans, green, broadly sword-shaped, curved, midrib evident, 69.0–82.9 × 5.5–7.5 cm, the basal leaves fibrous. *Flowering stems* with 7–10 branched that arise in the axillary leaves, 1- or 2 leaved subtend the flower on the branch, ca. 50.0 × 4.5 cm. *Spathes* 2 or 3, green, lanceolate, ca. 2 cm, 3- or 4- flowered, seldom 5, apex acuminate. *Flowers* blueish violet, 11.5–12.8 cm in diam.; *pedicel* 1.5–3.0 cm, perianth tube slender, ca. 1.5 cm; outer segments mottled darker around conspicuous, yellow, irregularly toothed crest, broadly ovate, 6.8–7.3 × 5.2–5.5 cm; inner segments spreading horizontally at anthesis, elliptical, 5.5–6.2 × 3.9–4.2 cm. *Stamens* ca. 2 cm; anthers bright white without pollen. *Style branches* pale bluish violet, 4–5 cm, feathery apex, terminal lobes fimbriate. *Ovary* cylindrical, ca. 2 cm. Flowering season, March–April. Sterile.

##### Etymology.

The new hybrid is named for the large flower.

##### Distribution and habitat.

Iris×ampliflora was collected from the Qingyang Town, Fuling District, Chongqing, China (29°31'40.8"N, 107°12'54"E). With complicated mountainous topography, the Qingyang Town is located in the range of the Dalou Mountains with an average altitude of 750 m. There were about 10 clones each with 6–10 individuals in the population of I.×ampliflora, covering an area of 200 m^2^. Plants of I.×ampliflora grow well on roadsides of subtropical mixed evergreen deciduous broad-leaved forest in full-sun and partly-shaded environments at an altitude of about 650 m. The lowest and highest temperature of the original site are about -5 °C and 38 °C, respectively.

##### Phenology and reproductive characteristics.

Iris×ampliflora blooms in March to April in Chongqing, and it blooms in April in Shanghai. It is evergreen. No fruits have been observed, but it can reproduce vegetatively.

##### Preliminary conservation status.

Only one population of I.×ampliflora was found by our investigation in Qingyang Region and there are risks of disturbance by human activities. I.×ampliflora is currently cultivated in the conservation nurseries of Shanghai Botanical Garden and the Flower Fragrance Horticulture Limited Company in Chongqing.

##### Other *Iris* species examined.

***I.japonica*** Chongqing Municipality, WUK0495843; PE01012482; PE01012483; PE01012489; PE01012492; IMC0013795. ***I.confusa*** Chongqing Municipality, CDBI0169691, IMC0013989, IMC0013998; IMC0014000; IMC0014010. ***I.milesii*** Yunnan Province, LBG 00106670; LBG00106671; KUN0360444. ***I.wattii*** Chongqing Municipality, KUN0360622. Mount Emei, Sichuan Province, CSH0086611. Liangshan Prefecture, Sichuan Province, PE01013840. ***I.tectorum***, Chongqing Municipality, IBSC0629040; IBSC0629027; CDBI0169658.

## Discussion

With the characters of maternal inheritance in cpDNA genes, *I.japonica* was most likely postulated as maternal parent of the hybrid I.×ampliflora because these two cluster as sister taxa in a clade in the phylogenetic tree. It is difficult to find the actual maternal parent of I.×ampliflora because the chromosome number of *I.japonica* is variable, 2*n* = 28, 30, 31, 32, 33, 34, 35, 54 and 55 (British Iris Society Species Group 1997). However, the chromosome number of the paternal parent of the hybrid can be speculated, 2*n* = 34, 31, 30, 29, 28, 27, since I.×ampliflora has 31 chromosomes. Thus, the possibilities about the parentage of I.×ampliflora are: *I.japonica* (2*n* = 34) × *I.tectorum* (2*n* = 28) or *I.japonica* (2*n* = 32) × *I.wattii* / *I.confusa* (2*n* = 30).

Furthermore, the parents of I.×ampliflora can be deduced according to morphological features. The hybrid has aerial stems (mean length = 27.0 ± 2.7 cm, n = 10). Without an aerial stem, *I.tectorum* cannot be the paternal parent. *Iriswattii* and *I.confusa* both have aerial stems (*I.wattii*, mean length = 67.2 ± 19.9 cm, n = 10; *I.confusa*, mean length = 30.3 ± 9.3 cm, n = 10). However, the leaf surface of I.×ampliflora has no waxy coat; it is dull and ruffled similar to that of *I.wattii*. The leaf surfaces of *I.confusa* and *I.japonica* have a waxy coat, glossy and smooth. Thus, compared with *I.confusa*, *I.wattii* is possibly more likely to be the paternal parent of I.×ampliflora.

Though *I.japonica* and I.×ampliflora are clustered into one clade, there are 22 mutations between the sequences of these two species. Compared with leaves (length 45–51 cm and width 3–4.5 cm) and flowers (diam. 4–5 cm) of *I.japonica*, I.×ampliflora has larger leaves (length 68–80 cm and width 6–7 cm) and larger purple flowers (diam. 11–13 cm). It cannot be determined which population of *I.japonica* it is derived from for the maternal parent since intraspecific chromosome numbers are variable. Thus, the sequence divergence could not reflect the real evolutionary distance between *I.japonica* and I.×ampliflora. The evolution of I.×ampliflora is in need of further study.

## Supplementary Material

XML Treatment for Iris × ampliflora

## Appendix 1

**Table A1.** Genbank accession of two chloroplast fragments for nine *Iris* species (Mathew 1981; Goldblatt and Manning 2008) in the study.

Species	Collect location	Collector	Genbank accession	
			*mat*K	*ndh*F
I.×ampliflora	Shanghai Botanical Garden	Yue-E Xiao	MW203044	MW203053
*I.anguifuga*		Yue-E Xiao	MW203045	MW203054
*I.confusa*		Yue-E Xiao	MW203046	MW203055
*I.domestica*		Yue-E Xiao	MW203043	MW203052
*I.henryi*		Yue-E Xiao	MW203047	MW203056
*I.japonica*		Yue-E Xiao	MW203048	MW203057
*I.proantha*		Yue-E Xiao	MW203049	MW203058
*I.tectorum*		Yue-E Xiao	MW203050	MW203059
*I.wattii*		Yue-E Xiao	MW20305	MW203060
